# Complete genome sequence of *Bacillus subtilis* SNUY1, a novel strain enhancing antifungal activity in edible coatings

**DOI:** 10.1128/mra.01061-25

**Published:** 2025-12-23

**Authors:** Gwon Mo Yang, Na Yun Kim, Yohan Song, Donghwa Chung, Hyo Jin Kim

**Affiliations:** 1 Graduate School of International Agricultural Technology, Seoul National University 26725, Pyeongchang, Republic of Korea; 2 Institutes of Green Bio Science and Technology, Seoul National University 26725, Pyeongchang, Republic of Korea; Fluxus Inc., Sunnyvale, California, USA

**Keywords:** antifungal activity, *Bacillus subtilis *SNUY1, *Botrytis cinerea*, edible coating, hybrid sequencing

## Abstract

*Bacillus subtilis* SNUY1, isolated from Korean fermented food, strongly inhibits *Botrytis cinerea*. Its 4.19 Mb genome comprises 4,164 protein-coding genes and 108 pseudogenes (4,272 CDSs) without plasmids and includes 33 antifungal-related genes. These genetic traits highlight its potential as a natural food additive to control mold growth on perishable produce.

## ANNOUNCEMENT

*Bacillus subtilis*, a gram-positive, endospore-forming bacterium, is valued for enzyme production and antifungal activity (1–3). Edible coatings incorporating this strain can slow deterioration, browning, and microbial contamination of fresh-cut produce, extending shelf life with a natural spoilage-control strategy (4–6).

We screened hundreds of beneficial bacteria isolated from traditional fermented foods in Gangwon-do, South Korea (38°04′31.4″N, 128°37′07.9″E). Among these, *B. subtilis* SNUY1, particularly from *Doenjang*, exhibited strong antifungal activity against *Botrytis cinerea*, a major fresh-cut spoilage agent (7). To evaluate the antifungal traits and industrial potential of the isolate, we performed whole-genome sequencing. Extended methods are available on protocols.io (8).

The *B. subtilis* strain, isolated as a single colony, was stored in glycerol stock and cultured in tryptic soy broth at 25°C with shaking for 24 h, then plated on tryptic soy agar and incubated at 30°C for 24 h. Genomic DNA from *B. subtilis* SNUY1 was extracted as described (9), and its quantity and purity were assessed.

Whole-genome sequencing of high-quality DNA was conducted by Macrogen (South Korea) using both PacBio Revio (PacBio, USA) and Illumina NovaSeq X Plus (Illumina, USA) systems. For PacBio long reads, a library was prepared with the SMRTbell prep kit 3.0 (PacBio, USA); high-molecular-weight DNA was sheared to ~7–12 kb using g-TUBE (Covaris, USA) and size-selected with a bead-based method. Sequencing on the Revio platform used the Revio SPRQ polymerase kit and Revio SMRT Cell tray with a 24-h movie, yielding 181,980 HiFi reads (1.35 Gbp; N50 = 8,919 bp). A short-read library with an average insert size of ~350–450 bp was prepared using the TruSeq Nano DNA High Throughput Library Prep Kit (Illumina, USA) and sequenced on the Illumina NovaSeq X Plus platform with 2 × 151 bp paired-end reads. Size selection was conducted using AMPure beads to isolate DNA fragments of the desired size, resulting in 16,374,820 reads and a total of 2.5 Gbp of sequencing data, with 96.6% of bases achieving a Q30 score or higher. Prior to assembly, Illumina short reads were quality filtered using Trimmomatic (v0.38) and assessed with FastQC (v0.11.7).

*De novo* assembly of PacBio Revio HiFi reads with SMRT Link Microbial Genome Analysis (v25.1.0.257715) produced a single 4,196,931 bp circular contig, which was polished with Illumina reads using Pilon v1.22 (10). Circularity was confirmed with SMRT Link v25.1 and Inspector v1.0.1; the chromosome was rotated to dnaA; mean coverage was 322×; and BUSCO v5.1.3 (bacteria_odb10) completeness was 100%.

Open reading frames (ORFs) and non-coding RNAs were predicted using Prokka (v1.14.6) (11), which integrates various tools such as Prodigal, RNAmmer, Aragorn, SignalP, and Infernal. Functional annotation of the predicted genes was performed using InterProScan (v5.34-73.0) (12) and eggNOG DB (v4.5) (13). Gene clusters associated with secondary metabolite production and polysaccharide degradation were additionally predicted using antiSMASH (v4.1.0).

**Fig 1:**
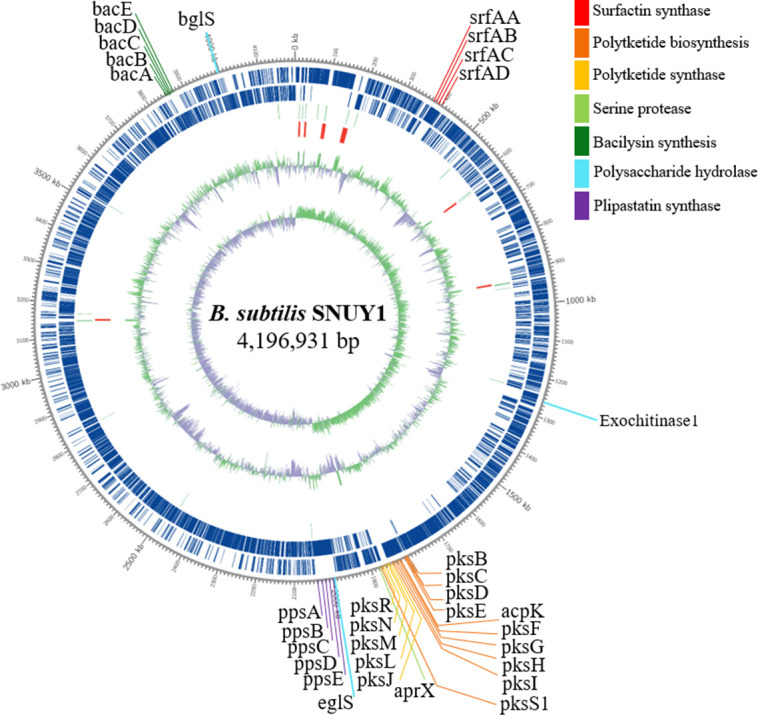
Complete chromosomal genome of *B. subtilis* SNUY1. Circos (v0.69-9) was used to render the circular genome map. On the forward and reverse strands appear on the outer ring; GC content and GC skew are shown on the inner rings. Thirty-three antifungal-related genes are highlighted along the rim.

The complete genome of the *B. subtilis* SNUY1 strain comprises a single, circular chromosome measuring 4,196,931 bp, with a G + C content of 43.7% (Fig. 1 and Table 1). The genome encodes 4,272 coding sequences (4,164 protein-coding and 108 pseudogenes) and a total of 4,393 genes, with no plasmids detected, as annotated by the NCBI Prokaryotic Genome Annotation Pipeline (PGAP v6.10). Fig. 1 shows 33 antifungal-related genes in *B. subtilis* SNUY1 that inhibit *B. cinerea*, supporting its application in fruit coatings.

**TABLE 1:** Assembly and genome features of *B. subtilis* SNUY1

Feature	*B. subtilis* SNUY1
Genome size (bp)	4,196,931
G + C content (%)	43.7
No. of contigs	1
N50 (bp)	4,196,931
Coverage (×)	322
Total genes	4,393
Total coding sequences (CDS)	4,272 (including 4,164 protein-coding and 108 pseudogenes)
tRNA	86
rRNA	30
ncRNAs (including tmRNA)	5
BUSCO completeness (%)	100

## Data Availability

The genomic data of *B. subtilis* SNUY1 were deposited in the NCBI under the BioProject accession number PRJNA1327643, the BioSample accession number SAMN51289187, the SRA accession number SRR35360082, and the GenBank accession number CP199918.
